# Effect of scaling on the invasion of oral microorganisms into dentinal tubules including the response of pulpal cells—an in vitro study

**DOI:** 10.1007/s00784-020-03705-7

**Published:** 2020-12-04

**Authors:** Alexandra Stähli, Alex S. J. Schatt, Miro Stoffel, Sandor Nietzsche, Anton Sculean, Reinhard Gruber, Barbara Cvikl, Sigrun Eick

**Affiliations:** 1 Department of Periodontology, School of Dental Medicine, University of Bern, Freiburgstrasse 7, 3010 Bern, Switzerland; 2 Center of Electron Microscopy, University Hospital Jena, Jena, Germany; 3 Department of Oral Biology, School of Dentistry, Medical University of Vienna, Vienna, Austria; 4 Department of Conservative Dentistry, Sigmund Freud University, Vienna, Austria

**Keywords:** Bacterial invasion into dentine, Bacterial penetration into dentinal tubules, Endodontic-periodontal lesion, Mixed species biofilm, Periodontal therapy, Proinflammatory response, Pulpal cells, Scaling

## Abstract

**Objectives:**

To investigate how scaling affects the penetration of microorganisms into dentinal tubules, how pulpal cells seeded into the pulp cavity respond to bacterial challenge, and how penetration and inflammatory response may depend on the bacterial composition.

**Materials and methods:**

Root canals of 102 extracted human teeth underwent shaping and cleaning. Half of the teeth were subjected to scaling and root planing, the other half remained untreated. Teeth were exposed to either *Streptococcus gordonii* and *Actinomyces oris* or *S. gordonii* and *Porphyromonas gingivalis* for 10 weeks. Bacterial invasion was assessed in a depth of 1 mm to the root surface. Human pulpal cells were seeded into the cavities to assess the expression of interleukin-8 (IL-8), monocyte chemoattractant protein-1 (MCP-1), and matrix metalloproteinase-3 (MMP-3) by real-time polymerase chain reaction and immunoassay.

**Results:**

The percentage of teeth with bacteria detected in dentine was higher when teeth received scaling than when they were untreated: 66.6% versus 44.4% when exposed to *A. oris*/*S. gordonii*, and 50% versus 25% when exposed to *P. gingivalis*/*S. gordonii* (*p* = 0.043). Scaling had no impact on IL-8 and MMP-3 expression in pulpal cells. *P. gingivalis*/*S. gordonii* caused higher levels of IL-8, MCP-1, and MMP-3 than *A. oris*/*S. gordonii* (*p* = 0.003, *p* = 0.011, *p* = 0.037).

**Conclusion:**

Scaling supports the penetration of bacteria into the dentine of extracted human teeth. *P. gingivalis* may affect the immune response in pulpal cells.

**Clinical relevance:**

Root surface debridement with hand instruments may facilitate bacterial penetration. Other kinds of mechanical instrumentation in this experimental setting should be investigated.

## Introduction

The tooth’s pulpal tissue together with the surrounding periodontal apparatus form one biologic unit, a continuum where alterations in one compartment may have an impact on the others. Therefore, also therapeutic interventions in one compartment potentially can exert broader effects. Mechanical instrumentation, the standard treatment of periodontitis, aims to eliminate hard and soft bacterial deposits on the root surface and results in improved clinical outcomes such as reduced bleeding on probing (BoP) and decreased probing pocket depth (PPD) [1, 2]. However, repeated or extensive scaling and root planning alters the surface of the root cementum and can thus have an impact on the pulpal tissue. Root cementum is a thin continuous mineralized tissue layer that forms the outer cover of the root and connects the periodontal ligament and the dentine [3]. It serves as a site of attachment for the Sharpey’s fibers and therefore plays a crucial role for tooth attachment [4]. When bacterial contamination and the immune host response spread towards the apex on the root cementum, the question arises whether bacteria, endotoxins, or cytokines are able to invade into the cementum layer.

As a matter of fact, subgingival mechanical instrumentation during active (i.e., nonsurgical and surgical) and supportive periodontal therapy results in the removal of more or less cementum which can eventually lead to exposure of dentinal tubules, pulp injury, or hypersensitivity [5]. Root substance removal by scaling and root planing with hand instruments was measured applying different known stroke forces. Substance removal per stroke varied between 6.8 and 20.6 micron depending on the force used. Interestingly, along 40 strokes, the mean force per stroke increased while the substance removal per stroke decreased resulting in a cumulative loss of 148.7 microns for low and 343.3 micron for high forces [6].

Periodontal disease and instrumentation of the root surface might influence pulpal tissues due to their close anatomical relationship: primarily through exposed dentinal tubules, lateral or accessory canals, and via the apical foramen. Once dentine tubules become exposed, microbial inflammation is potentially able to spread bi-directionally. As such, it is not surprising that the microbiota infecting the periodontium and the root canal systems are highly similar. Disease transmission and microbial similarity has been demonstrated by many studies [7–9] and is further supported by the similar cellular infiltrate in the pulpal and periodontal tissues [10]. Analysis of microbiomes of endodontic-periodontal lesions before and after root canal treatment shows a highly diverse microbial community with bacteria surviving root canal treatment. Hereby, dentinal tubules might serve as niches for bacterial survival. Endodontic bacteria either in monomicrobial or in polymicrobial aggregates were detected in the dentinal tubules of extracted infected teeth [11]. *Actinomyces* sp. is abundant in deep dentine lesions [12] and is found in endodontic infections [13]. Also for yeasts, in particular *Candida albicans*, it is possible to colonize the dentinal tubules system as well as the periodontal pocket [14]. Despite the similarities of the microbiota of the pulp and the periodontium, periodontal pockets show a higher diversity in species than in the root canal system [15, 16]. In endodontic samples of teeth affected by periodontitis periodontal pathogens including *Aggregatibacter actinomycetemcomitans*, *Porphyromonas gingivalis*, *Treponema denticola*, or *Tannerella forsythia* could be detected [17].

On the other side, periodontal inflammation can affect the vitality of the pulp. In periodontally compromised teeth without restorations and caries lesions, histological signs of pathology were found adjacent to accessory canals in 79% of teeth [18]. Further, a positive association between the severity of periodontal disease and histological changes in the pulp was detected [19]. More attachment loss was correlated with more pronounced signs of pathological changes [19]. There are, however, contradictory results concerning the effect of periodontal disease on the pulp. Clinical studies implied that periodontal disease only rarely results in pulp necrosis as was suggested before. Over a time period of 4 to 13 years, only few teeth with advanced periodontal disease experienced pulpal necrosis and needed root canal treatment [20]. On cellular level, only few to no inflammatory cells of periodontally involved roots were reported by Haskell et al. when resecting vital roots [21].

To date, evidence on bacterial challenges on the root surface after mechanical instrumentation and their impact on pulpal cells is scarce. The present study used a 10-week biofilm cultivation to determine (i) the impact of mechanical instrumentation on the penetration depth of bacteria from the root surface into dentine and (ii) the cellular response of pulpal cells seeded into the pulpal cavum of extracted scaled and native teeth that were exposed to two-species bacterial mixtures (i.e., *Streptococcus gordonii* with *Actinomyces oris* as well as *S. gordonii* with *P. gingivalis*), and (iii) differences between the two bacterial compositions.

## Material and methods

### Preparation of extracted teeth

**Fig. 1:**
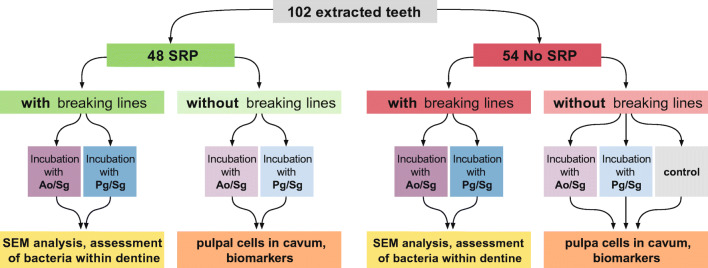
Flowchart of the experimental setting and the different groups of used teeth. SRP, scaling and root planning; Ao, *Actinomyces oris* MG1; Pg, *Porphyromonas gingivalis* ATCC 33277; Sg, *Streptococcus gordonii* ATCC 10558

A total of 102 extracted adolescent premolars or upper wisdom teeth were used. The teeth had been extracted over the last years at the School of Dental Medicine, Bern, and anonymously stored in 1% chloramine for a long time before storing in 0.9% w/v NaCl solution. The volunteers were informed about the use of their extracted teeth in research, and their oral consent was obtained. According to the guidelines, no previous approval from the Cantonal Ethical Committee, Bern, (KEK) was necessary as the teeth were categorized as “irreversibly anonymised.” Pulpal chambers and root canals were instrumented 1 mm short of the apical foramen using endodontic files and ProTaper Next instruments up to 40.06 (PTN; Dentsply Maillefer, Ballaigues, Switzerland). During instrumentation, the teeth were irrigated with 3 ml of NaOCl solution (Dakin’s solution, natrii hypochlorosi 3%; Hänseler, Swiss Pharma, Herisau, Switzerland). To remove remnants of the disinfecting solution, the teeth were stored in 0.9% w/v NaCl solution for two months with exchange of the solution every third day. Half of the teeth then received scaling and root planing with Gracey curettes (HuFriedy, Chicago, IL, USA) for 4 min by a periodontist. The apices of the teeth were closed with composite (Tetric Evo Flow A2, Ivoclar Vivadent, Schaan, Liechtenstein) and Syntac system (Ivoclar Vivadent, Schaan, Liechtenstein) in order to prevent apical leakage. Predetermined breaking lines in the longitudinal axis were produced with a diamond bur in half of the scaled and half of the native teeth. Figure 1 depicts the experimental setting and the groups of the extracted teeth. Pulpal chambers were then filled with a sterile cotton pellet and closed with a temporary material (Telio CS, Ivoclar Vivadent, Schaan, Liechtenstein) using a curing lamp (Bluephase, Ivoclar Vivadent, Schaan, Liechtenstein). The teeth were embedded in vertical direction in an empty pipette tip box using silicone for their fixation (Putty regular set, Provil novo Dose Refill, Heraeus Kulzer GmbH, Hanau, Germany). Before the experiments, they were autoclaved in the box half filled with distilled water for 20 min at 121 °C.

### Microbial penetration of roots of the teeth

The following microbial strains were used: *S. gordonii* ATCC 10558, *A. oris* MG1, and *P. gingivalis* ATCC 33277. Strains were cultivated on trypticase soy agar [22] plates (Oxoid, Basingstoke, GB) with 5% of sheep blood for 24 h at 37 °C (*A. oris* MG1 and *P. gingivalis* ATCC 33277 in anaerobic conditions). Thereafter, microorganisms were suspended in 0.9% w/v NaCl solution (OD600 nm = 1). Then, suspensions of *S. gordonii* were mixed 1:1 either with *A. oris* or with *P. gingivalis*, before adding to the nutrient broth ((Wilkins-Chalgren broth (Oxoid) with 10 μg/ml β-NAD (Merck KGaA, Darmstadt, Germany)) brain heart infusion broth Oxoid). Extracted teeth had been incubated in the nutrient media with bacteria for 10 weeks. For half of the teeth, *S. gordonii* and *A. oris* were used for the other half *S. gordonii* and *P. gingivalis*. The boxes containing the teeth were opened in a laminar air flow cabinet and the roots were inoculated with one of the two bacterial suspensions. The boxes were incubated in an anaerobic chamber at 37 °C for 10 weeks. Every week, new bacteria were added and bacterial growth in nutrient media was checked. Exchange of the nutrient medium was performed every 3 days.

### Cell culture

Dental pulpal cells (DPC) were harvested from the extracted donor teeth from patients who had given informed and written consent. As before, the volunteers were informed about the use of their extracted teeth in research and according to the guidelines, no previous approval from the Cantonal Ethical Committee KEK was necessary as the teeth were categorized as “irreversibly anonymised.” Cells growing out from dental pulp tissue were incubated in cell culture medium (DMEM, Invitrogen; Carlsbad, CA, USA ) with 10% fetal bovine serum (FBS, Invitrogen). As fibroblasts within the dental pulp cell population show a high proliferation rate, it can be assumed that the dental pulp cell population predominantly consists of fibroblasts. A total of three strains of pulp cells were established by explant cultures and fewer than 10 passages were used for the experiments. Cells were maintained in a humidified atmosphere of 5% CO_2_ and at 37 °C. The cell culture medium was exchanged every other day and the FBS was reduced to 1%, one day prior to the experiments. Cells were seeded at a concentration of 300,000 cells/cm^2^, i.e., 5000 cells into the pulpal chambers of the extracted teeth using FBS-free cell culture medium.

### Scanning electron microscopy

For scanning electron microscopy [23], the teeth were split in half by fracturing, fixed with 2.5% glutaraldehyde in cacodylate buffer for 60 min, then washed twice with cacodylate buffer. Following dehydration with ethanol in ascending concentrations (30%, 50%, 70%, 80%, 90%, 100%), specimen were critical point–dried using liquid CO_2_ and sputtered with a 10-nm layer of gold. Microscopical analysis was performed using a scanning electron microscope (ZEISS LEO-1530, Carl Zeiss, Oberkochen, Germany) at an acceleration voltage of 8 kV.

### Penetration of bacteria into dentin

For assessing the penetration depth of bacteria into dentin, the teeth were longitudinally split in two halves. With a sterile rose bur, dentine specimens were collected out in a distance of about 1 mm to the root surface. The debris were placed into an Eppendorf tube filled with 100 μL of Wilkins-Chalgren broth with NAD and distributed on TSA plates with an inoculating loop. After anaerobic incubation for 7 days, bacterial growth was recorded.

### RT-PCR and immunoassay

mRNA expression of interleukin (IL)-8, monocyte chemoattractant protein 1 (MCP-1 = CCL2), and matrix metallo-protease (MMP)-3 were quantified in pulpal cells. RNA extraction was performed using the innuPREP Mini kit (Analytic Jena, Jena, Germany) and cDNA was generated from 100 ng total RNA using the GoScriptTM Reverse Transcription System (Promega, Madison WI, USA) according to the manufacturer’s instructions. Thereafter, real-time PCR using GoTaq® qPCR Master Mix (Promega) with respective primers (IL-8 fwd.: 5′-CAC TGC GCC AAC ACA GAA AT-3′, rev.: 5′-TGG CCC TTG GCC TCA ATT TT-3′, accession # BC013615.1; MCP-1 fwd.: 5′-GAT CTC AGT GCA GAG GCT CG-3′, rev.: 5′-TTT GCT TGT CCA GGT GGT CC-3′, accession # S71513.1; MMP-3: 5′-CAC CAG ATT TGC CAA AAG ATG CT-3′; 5′-TTG AGA CAG GCG GAA CCG AG-3′, accession # NM_002422.5; GAPDH [24]) was performed according to the manufacturer’s instructions. Amplification was performed with the QuantStudio 3 Real-Time PCR System (Thermo Fischer, Waltham, MA, USA). Relative gene expression was calculated with the delta delta CT method.

### Statistical analysis

Statistical analysis was based on the Kruskal-Wallis test and Mann-Whitney *U* test. For contingency tables, Fisher’s exact test was used. Analyses were performed using SPSS 26.0 (IBM Corporation, New York, NY, USA). Significance was set at *p* < 0.05.

## Results

From the 102 teeth at the beginning, only 91 could be included in the final analysis. In part, leakages occurred and these teeth had to be removed from final analysis.

### Invasion of mixed cultured strains

**Fig. 2:**
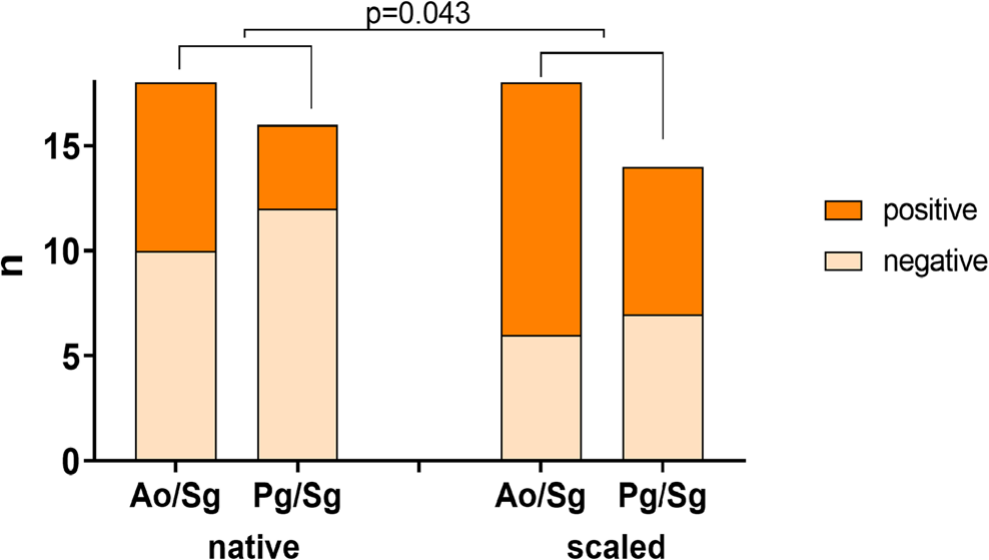
Dentine samples tested positively for invasion of mixed cultured strains (*Actinomycis oris* MG1/*Streptococcus gordonii* ATCC 10558 (Ao/Sg), *Porphyromonas gingivalis* ATCC 33277/*S. gordonii* ATCC 10558 (Pg/Sg)) in a depth of about 1 mm to the surface

Both bacterial mixtures were able to penetrate into dentine. For *A. oris*/*S. gordonii* as well as *P. gingivalis*/*S. gordonii*, scaled teeth showed a trend for a higher percentage of teeth with bacteria infected dentine compared to native teeth (Fig. 2). In the scaled group, 66.6% of the teeth exposed to *A. oris*/*S. gordonii* showed bacterial penetration through dentine, whereas in the native group, only 44.4% showed bacteria within dentine. For the teeth exposed to *P. gingivalis*/*S. gordonii*, 50% of the scaled teeth were positive versus 25% in the native group. When taking all the scaled teeth together and comparing them with the non-scaled teeth, the difference reached statistical significance (*p* = 0.043).

### Scanning electron micrographs

**Fig. 3:**
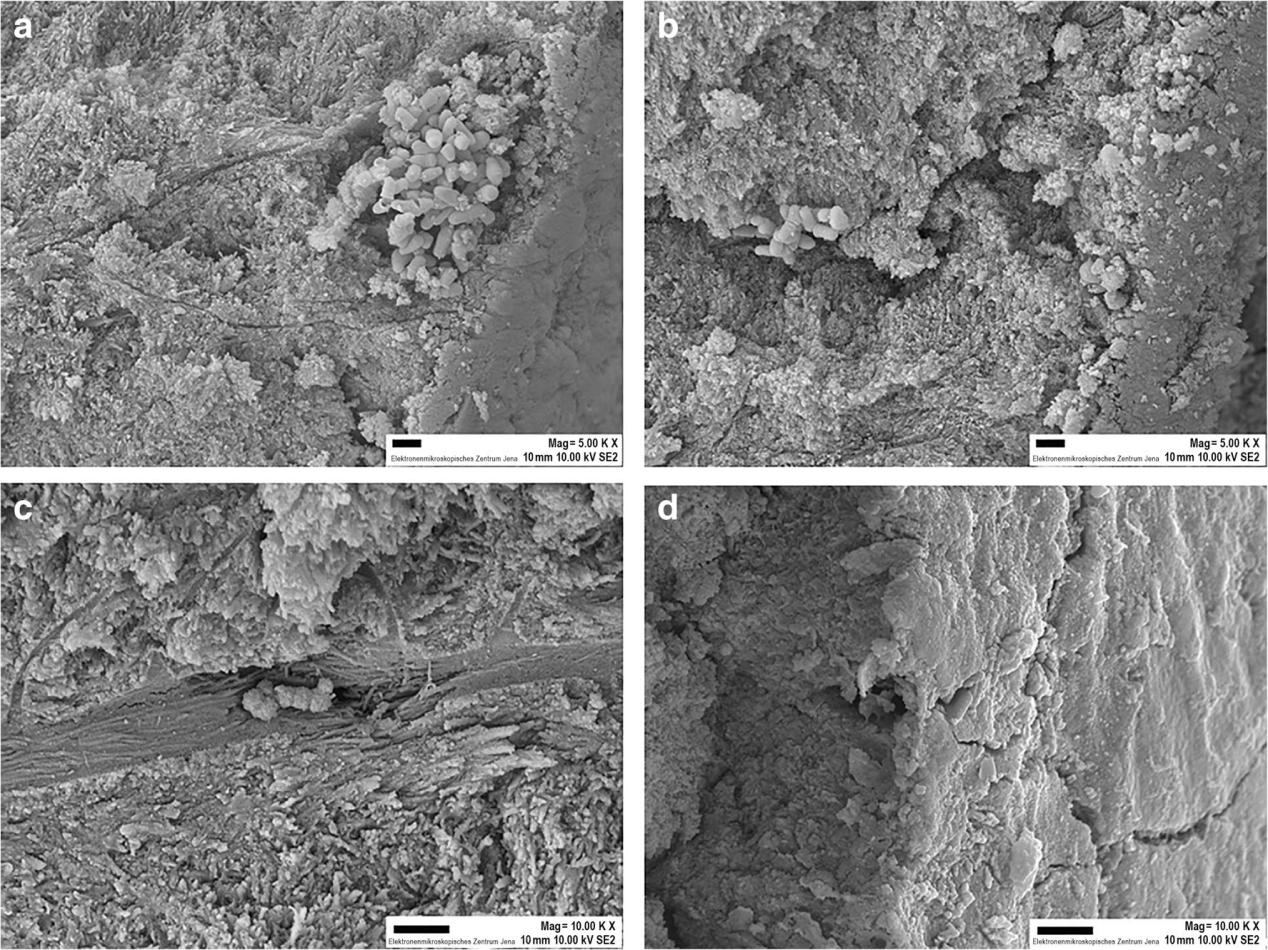
SEM images of fracture surfaces of root dentine revealing the infiltration of bacteria after contamination with *Actinomycis oris* MG1/*Streptococcus gordonii* ATCC 10558. **a**, **b**, **c** Scaled teeth: bacteria are located in high numbers in the irregularities close to the outer surface (**a**), they are able to penetrate into depth (**b** and **c**). **d** Native teeth. Only a few bacteria are attached to the outer surface. Magnification (**a**, **b**) 5.000 ×, (**c**, **d**) 10.000 ×. Scale bar = 1 μm

**Fig. 4.**
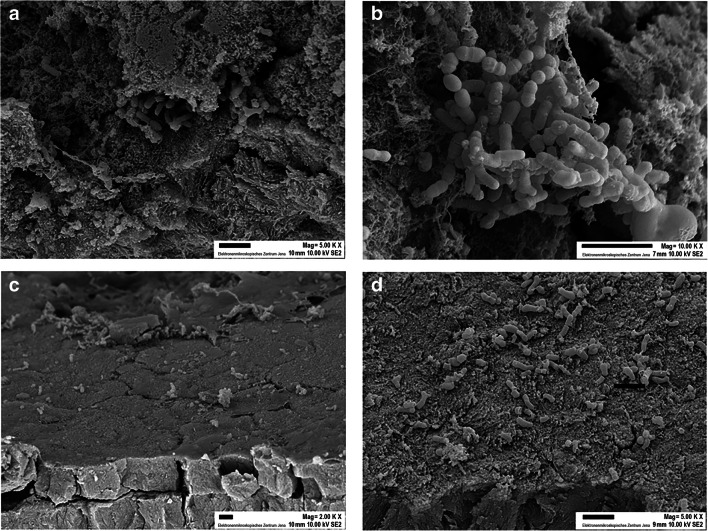
SEM images of fracture surfaces of root dentine revealing the infiltration of bacteria after contamination with *Porphyromonas gingivalis* ATCC 33277/*Streptococcus gordonii* ATCC 10558. **a**, **b** Scaled teeth: bacteria are located in high numbers in the irregularities at the outer surface. **c**, **d** Native teeth display an intact layer of cementum with only a few attached bacteria. Magnification (**a**, **d**) 5.000 ×, (**b**) 10.000 ×, (**c**) 2.000 ×. Scale bar = 2 μm

To visualize the results of the cultures, we also examined SEM images. At the scaled teeth, high amounts of bacteria were found in the surface irregularities while the native teeth showed only few bacteria attached to their surface. This was observed for both bacterial mixtures *A. oris*/*S. gordonii* (Fig. 3) and *P. gingivalis*/*S. gordonii* (Fig. 4). A few bacteria are visible deeper within the dentinal tubules.

### mRNA expression of inflammatory mediators

**Fig. 5:**
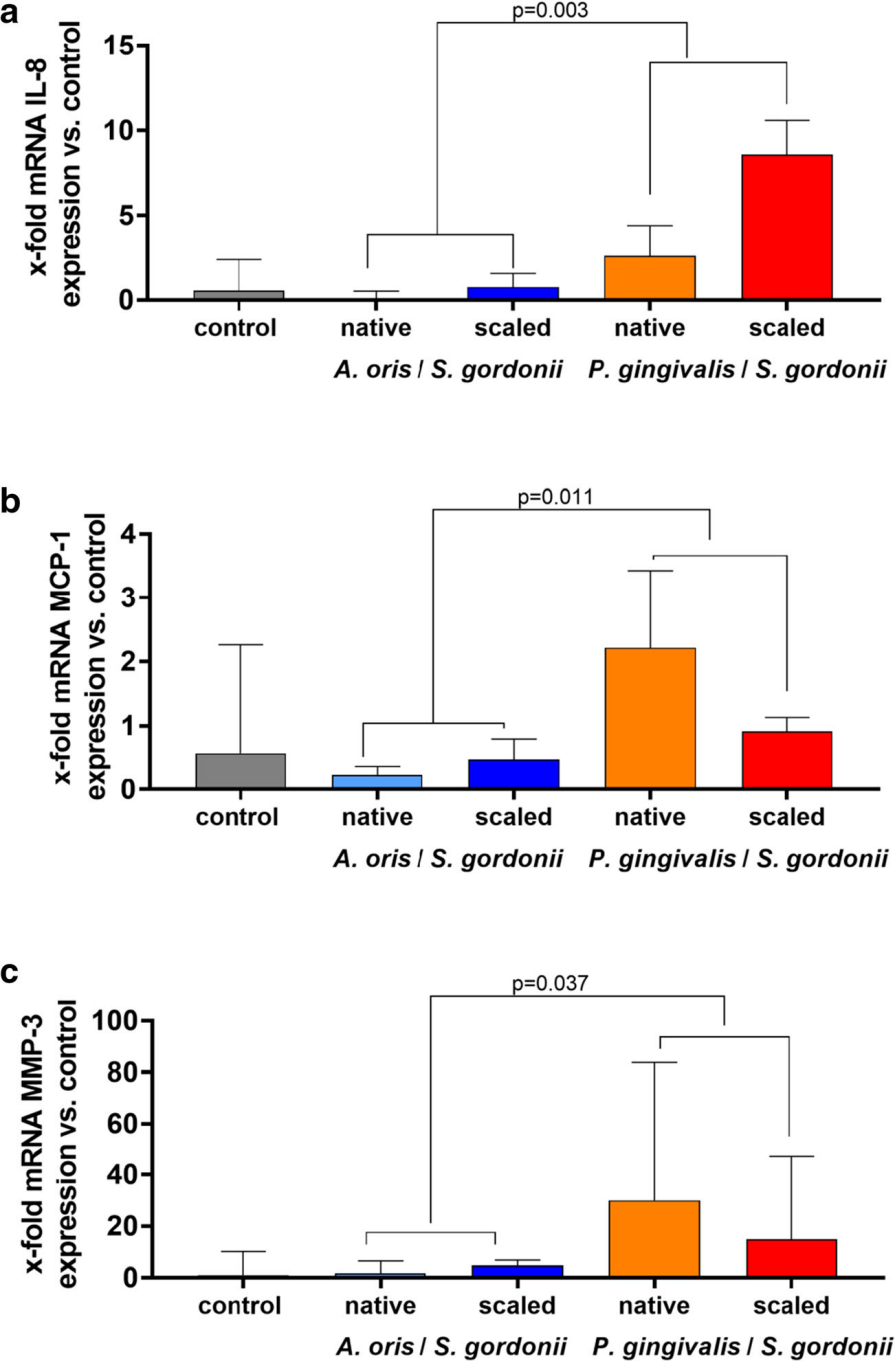
Interleukin (IL)-8 (**a**), monocyte chemoattractant protein 1 (MCP-1) (**b**), and matrix metallo-protease (MMP)-3 (**c**) mRNA expression in pulpal cells seeded into pulpal cavities after the teeth had been exposed to mixed biofilms for 10 weeks. Medians and interquartile ranges are presented

To determine the effect of scaling and root planing on proinflammatory cytokine expression, pulpal cells were seeded into the extracted and biofilm exposed roots. Compared to non-contaminated controls and non-treated contaminated teeth, the scaled teeth did not show increased expression of any mediator. However, when comparing the bacterial mixtures (independent whether the teeth were scaled or not), there was a higher level of IL-8, MCP-1, and MMP-3 mRNA expression when the teeth were exposed to *P. gingivalis* mixed with *S. gordonii* in comparison with the mixture *A. oris*/*S. gordonii* (*p* = 0.003, *p* = 0.011, *p* = 0.037) (Fig. 5).

## Discussion

In this in vitro study, we assessed the impact of mechanical instrumentation first on the penetration of bacteria into dentinal tubules and second on cellular responses of pulpal cells. The intent was to simulate the clinical exposure of root dentin either scaled or native to bacterial biofilm. For this purpose, the extracted adolescent premolar and upper third molar teeth were incubated in a two-species bacterial biofilm for 10 weeks. The major findings are that the instrumentation of the teeth affected the penetration of bacteria into dentine and furthermore that the expression of tested inflammatory mediators of pulpal cells was influenced by the bacterial species however not by instrumentation.

Particular emphasis in this study was set on the transmission of bacteria through dentine and the assumption that dentinal tubules opened by scaling and root planing are more patent for bacteria and their toxins. In periodontally diseased teeth, it has been shown that most bacteria are located in the outer 300 μm of dentinal tubules [25]. Here, the teeth were continuously incubated for a time period of 10 weeks to allow bacteria to spread into dentinal tubules. Based on our experimental approach, we discovered more bacterial penetration after mechanical instrumentation. Bacteria were observed within the root dentine for both two-species biofilms. Of note, the adolescent teeth generally show larger tubules while older teeth are more likely to exhibit signs of obliteration and sclerosis. This might have facilitated bacterial transmission. Co-culture of two bacterial species was chosen because co-culture of bacteria is closer to the in vivo situation as it is for example in periodontal disease. Furthermore, it has been shown that penetration of bacteria into dentinal tubules depends on bacterial species and on the complexity of the biofilm, whereby single species showed a different penetration pattern than co-culturing of bacteria [26]. In that study, the penetration of bacteria into dentine originated from the root canal. Penetration into the inner and middle third of the root dentin was detected for *S. gordonii*, *P. gingivalis*, and *A. oris*. Interestingly, when *P. gingivalis* and *A. oris* were co-cultured with *S. gordonii*, penetration until the outer third has been observed for the combination with *A. oris* but not *P. gingivalis*. [26]. In the present study, there might be by trend more penetration for *A. oris*/*S. gordonii* than for *P. gingivalis*/*S. gordonii*, but the results failed statistical significance. Others showed that the recognition of streptococcal cell wall-anchored polypeptides of the antigen I/II family accounts for the ability of *P. gingivalis* to invade dentinal tubules when in co-culture with *S. gordonii* but not with *Streptococcus mutans* [27]. This interplay of bacterial co-aggregation might play a role in the etiopathology of endodontic-periodontal lesions.

SEM images confirmed the penetration of bacteria into the dentinal tubules from the root surfaces. Further, there was a large number of bacteria visible in irregularities at the root surface after scaling. Scaling increases in vitro surface roughness of root surfaces which is accompanied by higher adhesion of *Streptococcus sanguinis* [28]. Smoother surfaces and less bacterial attachment in comparison with scaling are observed after ultrasonication [28].

Although histology has shown differences in pulpal tissue of teeth with moderate-to-severe chronic periodontitis [19], we found no differences in the gene and protein expression of proinflammatory cytokines of pulpal cells between the two groups (native vs scaled). However, when comparing the different bacterial mixtures, irrespective of the two groups of teeth, *P. gingivalis*/*S. gordonii* induced a significantly higher mRNA expression of all inflammatory mediators than *A. oris*/*S. gordonii* did. This effect might be related to gingipains. It can be assumed that gingipains can penetrate into the pulpal chamber. Already Bergenholtz and Lindhe suggested a pathway of communication between dentinal tubules, the periodontium, and the pulp [29]. In an animal model, it was shown that application of bacterial plaque to dentin readily caused pulpal inflammation, while occlusion of exposed dentin had a protective effect to the pulp [30, 31].

Gingipains have been shown to regulate the expression of MMPs and of tissue inhibitors of metalloproteinases (TIMPs) in oral fibroblasts and epithelial cells thus contributing to tissue destruction [32]. The Arg-gingipains enhance mRNA of IL-8 in gingival fibroblasts [33]. Further, challenging of human gingival fibroblasts with live *P. gingivalis* resulted in an increase of IL-8 and MCP-1 mRNA expression [34]. Both IL-8 and MCP-1 are important chemoattractant cytokines [35]. For MMP-3, an anti-inflammatory function in pulp infection is suggested [35], expression might be mediated also via IL-1[36].

Some limitations need to be considered. First, leakage at the apexes or accessory canals might have concealed possible differences among groups. Second, a healthy pulp is a complex tissue consisting of a panoply of cells. Third, the non-vital extracted teeth present different physical characteristics such as for example the redox potential and this might have severe consequences of bacterial transmission through dentinal tubules. Fourth, pulpal cells were not seeded into the pulpal chambers until the end of 10 weeks of biofilm incubation. And finally, it must be considered that in the clinical situation of a vital tooth, various defense mechanisms against a possible penetration of bacterial toxins or bacteria are present. This involves a physiological pulpal tissue pressure resulting in an outward flow through the dentinal tubules. When bacterial toxins reach the dental pulp, an inflammatory process occurs; this will initiate the deposition of tertiary dentine in the tubules and increase the above mentioned tissue pressure. Thus, the clinical implication of these findings might be taken with caution and requires future research.

Our study provides novel insights into the effects of root instrumentation on pulpal cell response when exposed to bacterial biofilm. Our experiments further indicated that penetration of bacteria through the dentine was more observed in the scaled group than in the group with the native teeth. Since the teeth were endodontically instrumented, we assume that in root canal–treated teeth, similar penetration patterns might be observed. SEM images showed a more pronounced colonization of bacteria at the surfaces of the scaled teeth, in particular in irregularities. Therefore, it has to be pointed out that a smooth root surface might be crucial in preventing massive bacterial adherence. In this respect, other kinds of mechanical instrumentation in this experimental setting should be investigated.

Previously, cementum of periodontally diseased root surfaces was believed to harbor bacteria and bacterial endotoxins, and therefore the removal of the “diseased cementum” was considered mandatory to allow periodontal healing [29, 37]. Later, it was shown that excessive removal of cementum is not necessary to achieve periodontal health [38]. Findings from an experimental study in dogs revealed that healing was similar whether or not the previously exposed root cementum had been removed [39]. Indeed, in vitro penetration of *Escherichia coli* lipopolysaccharide (LPS) into root cementum was detected neither in the periodontally healthy nor in the diseased teeth [40]. The binding of the LPS was limited to the root surface and furthermore seemed rather weak. It was further shown that 99% of LPS could be removed by relatively gentle procedures including washing or gentle brushing for 1 min with a slow rotating brush [41]. Consequently, the preservation of cementum was supported in order to enhance cell attachment and regeneration. But our data suggest that cellular response to bacterial challenge to the pulp depends not on the instrumentation but on the presence of periodontal pathogens. Following this, removal of the bacteria being associated with periodontal disease is crucial.

From a clinical point of view, the available data indicate that mechanical instrumentation of diseased root surfaces can be performed less aggressively with respect to cementum removal. We recently showed that compared to hand instrumentation, the application of ultrasonication and air-polishing with erythritol prevents from substance loss while providing a smooth surface with nearly no residual biofilm [42]. It might be of interest to investigate further these approaches regarding the endodontic-periodontal lesion.

## Conclusion

Root surface debridement with hand instruments may remove root cementum, which in turn facilitates bacterial penetration from the periodontal pocket and the oral cavity into the dentinal tubules system especially in the root canal–treated teeth where a cellular defense to bacterial penetration is lacking. In vital teeth, periodontal pathogens may influence pulpal response.
